# Phenethylamine in chlorella alleviates high-fat diet-induced mouse liver damage by regulating generation of methylglyoxal

**DOI:** 10.1038/s41538-021-00105-3

**Published:** 2021-07-23

**Authors:** Yifeng Zheng, Agustin Martin-Morales, Jing Wang, Masaki Fujishima, Eri Okumura, Kenji Sato

**Affiliations:** 1 Division of Applied Biosciences, Graduate School of Agriculture, Kyoto University, Kitashirakawa Oiwake-cho, Kyoto, Japan; 2 Research & Development Group, Sun Chlorella Co., Ltd., Osaka-cho, Kyoto, Japan

**Keywords:** Nutrition, Obesity

## Abstract

This study examined the effects of oral administration of water extract of chlorella (WEC) (100 mg/kg bodyweight) and phenethylamine (10 μg/kg bodyweight) on high-fat diet (HFD)-induced liver damage in mice. Phenethylamine significantly mitigated HFD-induced lipid oxidation (generation of malondialdehyde) and liver damage without markedly decreasing hepatic lipid accumulation. WEC exerted similar effects although with decreased efficacy. In addition, WEC and phenethylamine decreased the methylglyoxal levels and increased the glyceraldehyde 3-phosphate dehydrogenase (GAPDH) protein levels in the liver. Methylglyoxal is generated from substrates of GAPDH, dihydroxyacetone phosphate and glyceraldehyde 3-phosphate. These facts indicate that methylglyoxal triggers oxidation of accumulated lipid, which generates malondialdehyde and consequently induces liver damage. Suppression of generation of toxic aldehydes by WEC and phenethylamine was also confirmed by maintaining hepatic cysteine, highly reactive to aldehydes. Thus, trace amounts of phenethylamine alleviate HFD-induced liver damage by regulating methylglyoxal via increase of GAPDH.

## Introduction

*Chlorella pyrenoidosa*, a freshwater unicellular green alga, and its water extract have a long history of usage as food supplements. Various animal studies and clinical trials have reported that *C. pyrenoidosa* exhibits biological activities, such as anti-dyslipidemic and immunomodulatory activities upon oral administration^1,2^. Previously, we had identified phenethylamine (PHA) in the hot water extract of *C*. *pyrenoidosa* (WEC) at 10 μg/g dry matter and demonstrated that low dose (60 ng/g of feed) of PHA extends the lifespan of superoxide dismutase-1 gene (*Sod1)* mutant adults of *Drosophila melanogaster*^3^. This suggested that treatment with a low dose of PHA ameliorates oxidative stress in *D. melanogaster*, as SOD-1 plays a substantial role in the antioxidant system.

Trace amounts of PHA, which is classified as a monoamine, are detected in various types of food, such as cheese, chocolate, wine, fermented soy paste, and soy sauce^4–8^. In mammals, PHA is generated from phenylalanine through enzymatic decarboxylation. The oral supplementation of PHA (10–60 mg/day) with selegiline (monoamine oxidase-B inhibitor; 10 mg/day) alleviated depression in humans^9^. The administration of high doses of PHA (25–75 mg/kg bodyweight) promoted psychomotor dysfunction and decrease in striatal biogenic amines in mice^10^. Previously, we had reported the biological activity of trace amounts of PHA in *D. melanogaster*. However, the biological activity of trace amounts of PHA has not been examined in mammals. This study aimed to elucidate the beneficial effects of trace amounts of PHA in mammals.

A previous animal study demonstrated that the administration of dried powder of *C*. *pyrenoidosa* ameliorated high-fat diet (HFD)-induced dyslipidemia in rats^1^. HFD promotes hepatic damage in rats by increases in lipid deposition and lipid peroxidation and decreases in antioxidant enzyme activities in the liver^11,12^, which have been reported to induce hepatitis, especially non-alcoholic fatty liver disease (NAFLD)^13,14^. There is an increased prevalence of NAFLD in both advanced and developing countries. The enhanced lipid deposition-induced oxidative stress in the liver contributes to the pathogenesis of NAFLD^13,14^, whereas the liver contains potent antioxidant enzyme systems, such as the superoxide dismutase (SOD) and glutathione peroxidase (GPX) systems^15,16^. The mechanism underlying increased oxidative stress in the fatty liver has not been elucidated. Recently, methylglyoxal was reported to increase intracellular oxidative stress^17–19^. Methylglyoxal can be generated from metabolites of glucose and fructose, such as dihydroxyacetone phosphate and glyceraldehyde 3-phosphate^20^, which are metabolized by glyceraldehyde 3-phosphate dehydrogenase (GAPDH). In addition, methylglyoxal strongly reacts with the sulfhydryl, amino and guanidyl groups in amino acids and proteins^21,22^. Methylglyoxal-derived advanced glycation end products (AGEs), promotes the progression of steatosis to NAFLD and non-alcoholic steatohepatitis (NASH)^23^. Various studies have examined the inhibitory effect of food compounds on the formation of toxic AGEs using in vitro and in vivo system^24,25^. However, the administration of food compounds cannot effectively inhibit the generation of methylglyoxal.

This study aimed to examine the effects of trace amounts of PHA on HFD-induced oxidative stress and liver damage in mice. We found that PHA suppressed the peroxidation of lipids accumulated in the liver by decreasing the generation of methylglyoxal via increasing GAPDH in the liver.

## Results

### Effect of HFD feeding on mice

**Fig. 1:**
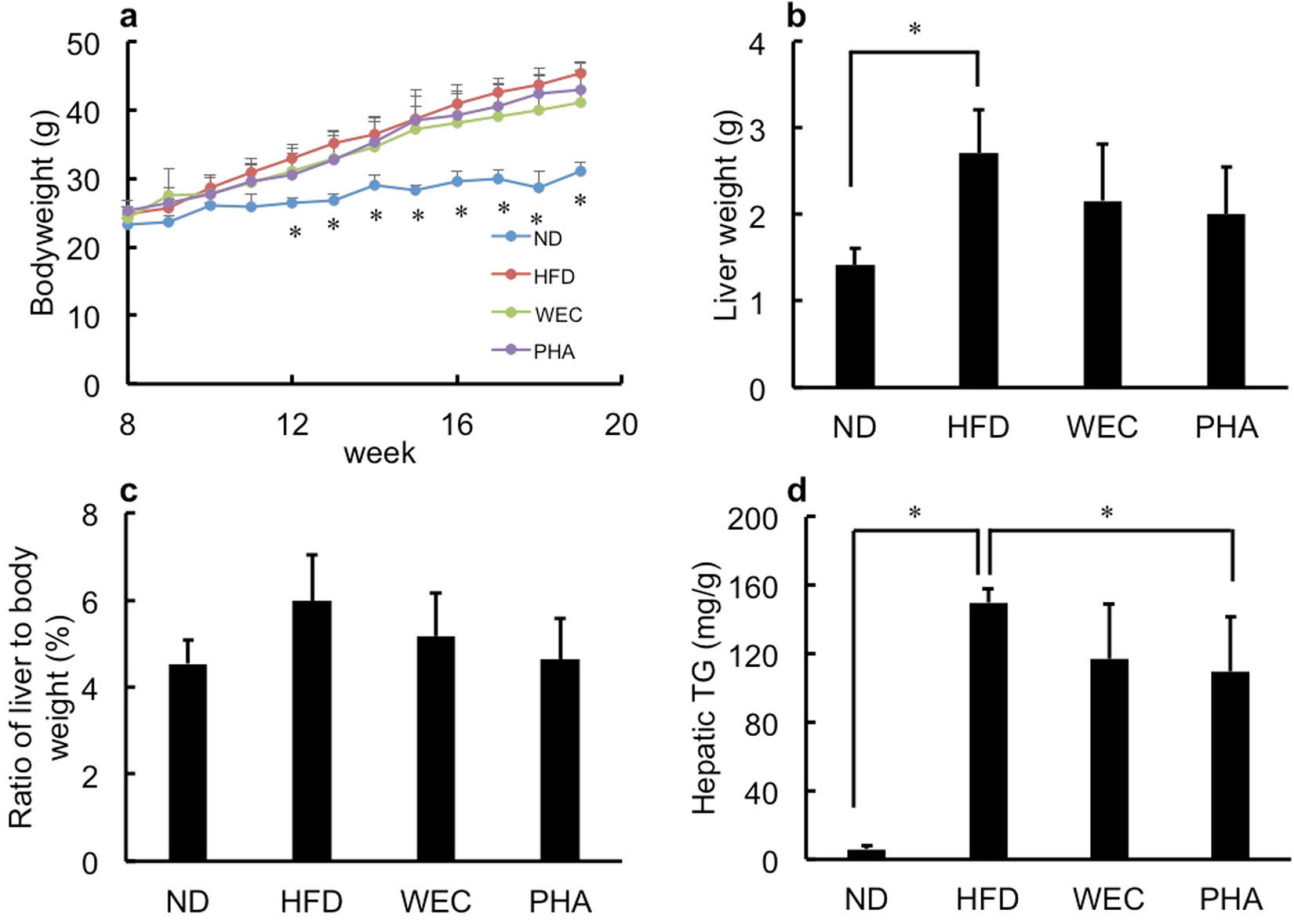
The effects of WEC and PHA on bodyweight and liver wight. Bodyweight change (**a**), liver weight (**b**), ratio of liver weight to bodyweight (**c**), and hepatic triglyceride levels (**d**) in the normal diet-fed (ND), high-fat diet-fed (HFD), *Chlorella pyrenoidosa* water extract-treated (WEC), and phenethylamine-treated (PHA) groups. The WEC and PHA groups were fed on HFD and administered with WEC (100 mg/kg bodyweight) and PHA (10 μg/kg bodyweight), respectively. Data are represented as mean ± standard deviation (*n* = 6). The means were compared using analysis of variance, followed by Dunnett’s test. *p < 0.05 vs. HFD group.

**Fig. 2:**
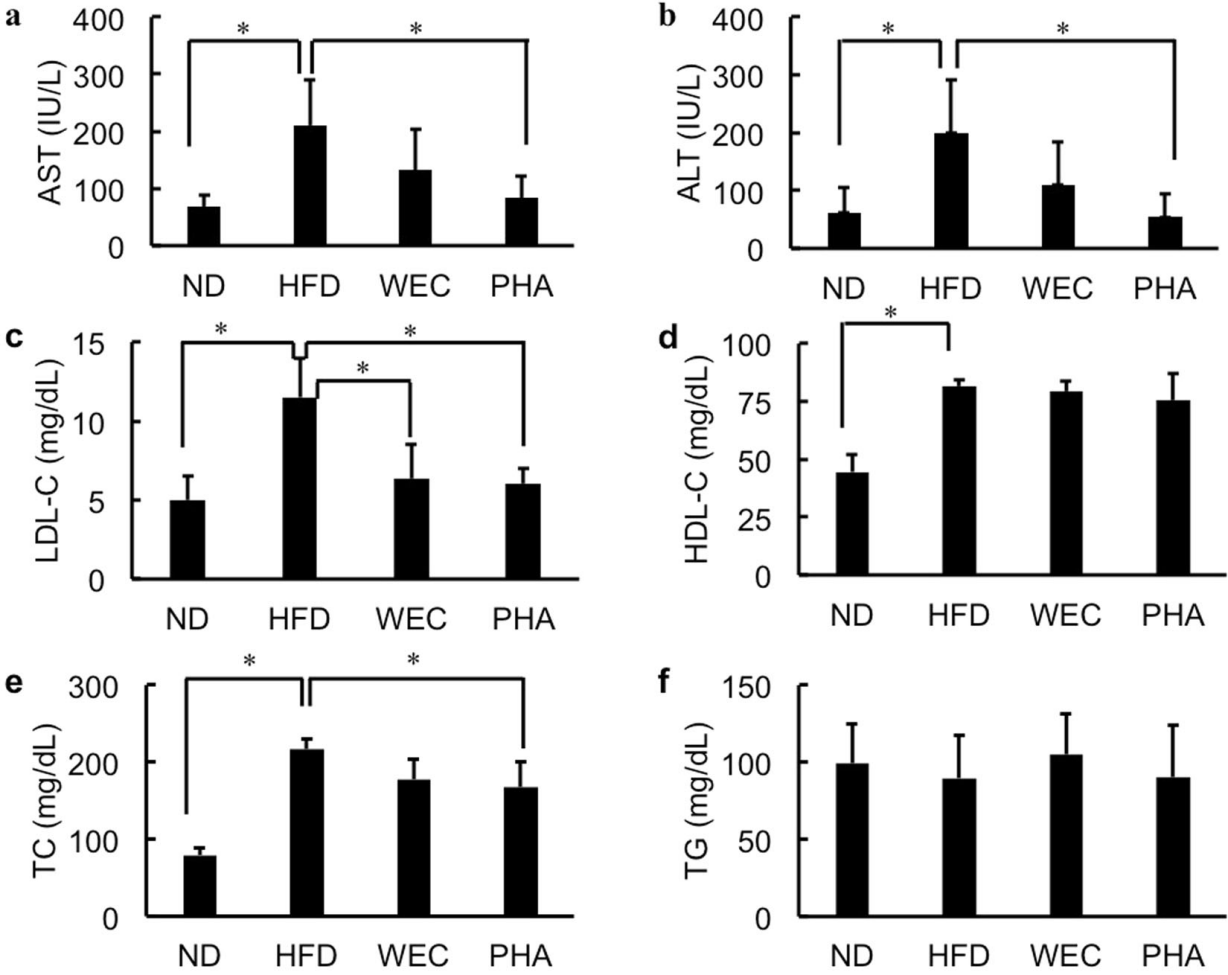
The effects of WEC and PHA on plasma biochemical parameters. Plasma aspartate aminotransferase (AST) and alanine aminotransferase (ALT) activities (**a**, **b**) and the levels of low-density lipoprotein-cholesterol (LDL-C; **c**), high-density lipoprotein-cholesterol (HDL-C; **d**), total cholesterol (TC; **e**), and triglyceride (TG; **f**) in the normal diet-fed (ND), high-fat diet-fed (HFD), *Chlorella pyrenoidosa* water extract-treated (WEC), and phenethylamine-treated (PHA) groups. Data are represented as mean ± standard deviation (*n* = 6). The means were compared using analysis of variance, followed by Dunnett’s test. *p < 0.05 vs. HFD group.

**Fig. 3:**
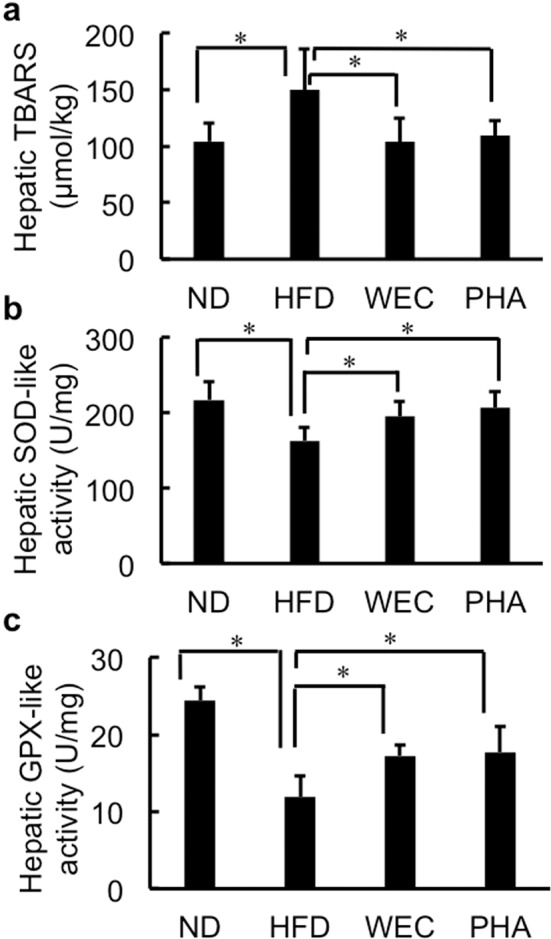
The effects of WEC and PHA on oxidative stress in liver. Thiobarbituric acid reactive substances (TBARS); (**a**) values (**a**), superoxide dismutase (SOD)-like activity (**b**), glutathione peroxidase (GPX)-like activity (**c**) in the liver of the normal diet-fed (ND), high-fat diet-fed (HFD), *Chlorella pyrenoidosa* water extract-treated (WEC), and phenethylamine-treated (PHA) groups. Data are represented as mean ± standard deviation (*n* = 6). The means were compared using analysis of variance, followed by Dunnett’s test. *p < 0.05 vs. HFD group.

Induction of NAFLD by a high-fat diet in the C57BL/6 strain is widely used among mice^26^. As shown in Figs. 1 and 2, HFD feeding induced obesity, fatty liver, liver damage, and increase of plasma low-density lipoprotein-cholesterol (LDL-C). Furthermore, increased lipid peroxidation and decreased antioxidant enzyme activities (SOD and GPX) were observed (Fig. 3).

### Effects of WEC and PHA on bodyweight and liver weight

The total calorie intake was similar among the HFD, WEC, and PHA groups. However, the total calorie intake in the HFD, WEC, and PHA groups was higher than that in the normal diet (ND) group (Supplementary Fig. S1). The addition of WEC and PHA into drinking water did not affect water intake (Supplementary Fig. S1). The administration of WEC and PHA did not significantly affect bodyweight and liver weight of HFD-fed mice (Fig. 1a, b). The ratio of liver weight to bodyweight was the highest in the HFD group (Fig. 1c). However, the ratio of liver weight to bodyweight was not significantly different between all groups (including ND group). The ratio of liver weight to bodyweight in PHA groups was lower than that in the HFD group (*p* < 0.1; Fig. 1c).

### Effects of WEC and PHA on liver and blood biochemical parameters

The administration of PHA significantly but slightly decreased liver TG (*p* < 0.05), while WEC did not significantly affect liver TG (Fig. 1d). PHA also significantly mitigated the HFD-induced enhanced plasma AST and ALT activities (*p* < 0.05; Fig. 2a, b). WEC showed similar tendency without statistic difference. WEC and PHA significantly decreased plasma LDL-C levels without affecting HDL-C levels (*p* < 0.05; Fig. 2c, d). In addition, PHA also significantly decreased TC (*p* < 0.05; Fig. 2e). As shown in Fig. 2f, the plasma TG levels were not significantly different between the HFD and other groups (ND, PHA, and WEC).

### Effects of WEC and PHA on oxidative stress in the liver

The administration of WEC and PHA significantly mitigated the HFD-induced enhanced lipid peroxidation (*p* < 0.05; Fig. 3a) and significantly increased SOD-like and GPX-like activities in liver extract (*p* < 0.05; Fig. 3b, c). In contrast, low molecular weight compounds in the 75% ethanol-soluble fraction of the liver extract from all groups had negligible SOD-like activity (Supplementary Fig. S2).

### Effects of WEC and PHA on hepatic methylglyoxal levels

**Fig. 4:**
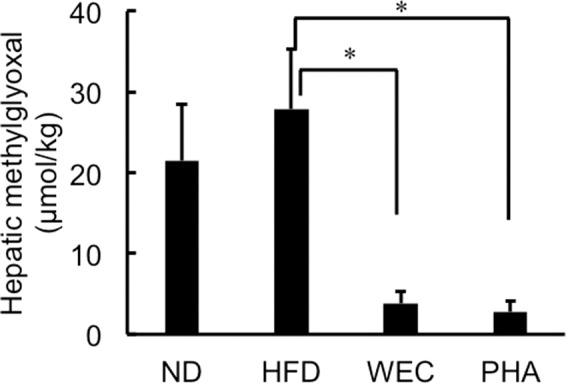
The effects of WEC and PHA on hepatic methylglyoxal levels. Hepatic methylglyoxal levels in the normal diet-fed (ND), high-fat diet-fed (HFD), *Chlorella pyrenoidosa* water extract-treated (WEC), and phenethylamine-treated (PHA) groups. Data are represented as mean ± standard deviation (*n* = 6). The means were compared using analysis of variance, followed by Dunnett’s test. *p < 0.05 vs. HFD group.

The hepatic methylglyoxal levels were not significantly different between the HFD and ND groups. In contrast, the administration of WEC and PHA significantly decreased hepatic methylglyoxal levels. (*p* < 0.05; Fig. 4).

### Effects of WEC and PHA on hepatic SOD1, GPX1, and GAPDH levels

**Fig. 5:**
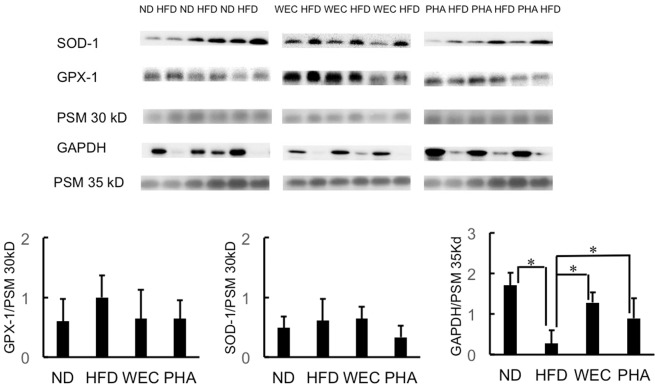
The effects of WEC and PHA on hepatic protein levels. Hepatic protein levels of SOD-1, GPX-1, and GAPDH in the normal diet-fed (ND), high-fat diet-fed (HFD), *Chlorella pyrenoidosa* water extract-treated (WEC), and phenethylamine-treated (PHA) groups. Data are represented as mean ± standard deviation (*n* = 6). The means were compared using analysis of variance, followed by Dunnett’s test. *p < 0.05 vs. HFD group. PSM, pre-stained marker.

In western blotting analysis, the housekeeping proteins, such as, GAPDH and β-actin, are used as loading controls. However, HFD feeding significantly decreased GAPDH levels (Fig. 5 and Supplementary Fig. S3) and tended to decrease β-actin (Supplementary Figs. S3 and S4). Among some mice in the HFD group, only a faint band of GAPDH was observed, whereas the pre-stained marker (35 kD) added to the sample exhibited similar band intensity. Thus, a pre-stained marker was used to normalize the expression levels of target proteins. As shown in Fig. 5, the SOD-1 and GPX-1 protein levels were not significantly different between the HFD and other groups (ND, PHA and WEC). On the other hand, the administration of WEC and PHA significantly increased GAPDH levels (*p* < 0.05; Fig. 5).

### Effect of WEC and PHA on hepatic cysteine levels

**Fig. 6:**
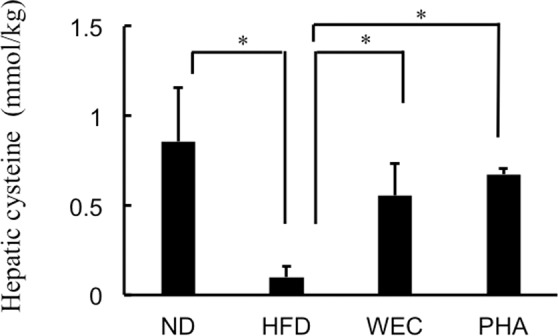
The effects of WEC and PHA on hepatic cysteine levels. Hepatic levels of cysteine in the normal diet-fed (ND), high-fat diet-fed (HFD), *Chlorella pyrenoidosa* water extract-treated (WEC), and phenethylamine-treated (PHA) groups. Data are represented as mean ± standard deviation (*n* = 6). The means were compared using analysis of variance, followed by Dunnett’s test. *p < 0.05 vs. HFD group.

The hepatic cysteine levels were not significantly different before and after dithiothreitol (DTT) treatment in all groups (Supplementary Fig. S5), which indicated that most of the soluble cysteine in the liver exists in the reduced form. As shown in Fig. 6, the hepatic cysteine level in the ND group was significantly higher than those in the HFD group (*p* < 0.05). The administration of WEC and PHA significantly increased the hepatic cysteine levels in the mice fed on HFD to the hepatic levels observed in the ND group (*p* < 0.05).

## Discussion

PHA promotes reactive oxygen species production in the yeast and plant (tobacco) cell culture systems^27,28^. Additionally, the oral administration or injection of PHA (> 25 mg/kg bodyweight) resulted in impaired behaviors in rodents^29,30^. These findings indicate that PHA exerts adverse effects at high doses, which may be through the induction of enhanced oxidative stress. Previously, we had demonstrated that trace amounts of PHA (60 ng/g of feed) extended the life span of a *Sod1* mutant adult of *D. melanogaster*^3^. However, the activity of such low dose of PHA has not been examined in mammals.　In this study, the treatment dose of PHA for mice was determined based on the content of PHA in the diet of *D. melanogaster* (60 ng/g in feed) and daily food intake of mice (~4 g/20 g bodyweight/day), which corresponded to 12 μg/kg bodyweight. Thus, the HFD-fed mice were administered with PHA at a dose of 10 μg/kg bodyweight through drinking water in this study.

As summarized in Table 1, PHA significantly mitigated HFD-induced lipid peroxidation and liver damage without markedly decreasing hepatic lipid accumulation. It is worthy to note that PHA significantly increased hepatic GAPDH and cysteine levels and decresed methylglyoxal levels. WEC exerted similar effects with decreased efficacy.

**Table 1:** Summary for responses by administration of WEC and PHA on mice fed high fat diet.

	WEC	PHA
Body weight	NS	NS
Liver TG	NS	slightly down
Plasma AST and ALT	NS	down
Plasma LDL-C	down	down
Plasma HDL-C	NS	NS
Hepatic TBARS	down	down
Hepatic SOD-like activity	up	up
Hepatic GPX-like activity	up	up
Hepatic SOD-1 level	NS	NS
Hepatic GPX-1 level	NS	NS
Hepatic GAPDH level	up	up
Hepatic methylglyoxal	down	down
Hepatic cysteine	up	up

NS: no significant change (*p* > 0.05).

Some antioxidant foods or compounds, such as ascorbic acid (30 mg/kg bodyweight)^31^, onion oil (100 mg/kg bodyweight) and/or garlic tablets (500 mg/kg bodyweight)^32^, and green tea extract (1% w/w) in diet^33^, as well as epigallocatechin gallate (1 g/L via drinking water)^34^, can attenuate hepatic oxidative stress in rodents induced by HFD. However, the doses of antioxidants or antioxidant foods used in previous studies were far higher than that of PHA used in this study (10 μg/kg bodyweight). PHA did not significantly increase SOD-like activity of low molecular weight compounds in liver. This indicated that the beneficial effects of such low dose of PHA do not involve direct antioxidant activity but involve the modulation of the expression of endogenous proteins. Actually, antioxidant enzyme activities (SOD-like and GPX-like activities) were significantly increased in the PHA group. Unexpectedly, WEC and PHA did not significantly affect the hepatic SOD-1 and GPX-1 protein levels. On the other hand, HFD feeding significantly decreased GAPDH protein levels, which is consistent with the previous stidies^35–37^. WEC and PHA significantly increased the hepatic GAPDH protein levels.

GAPDH, a key glycolytic enzyme, can decrease the levels of glyceraldehyde 3-phosphate and its isomer (dihydroxyacetone phosphate), which are precursors of methylglyoxal^20^. Beisswenger et al. demonstrated that the methylglyoxal level in the human blood red cells was positively and negatively correlated with dihydroxyacetone phosphate levels and GAPDH activity, respectively^38^. Methylglyoxal, a highly reactive short-chain aldehyde, induces oxidative stress in the cells^17–19^. In addition, methylglyoxal reacts with the sulfhydryl group in free cysteine and cysteinyl residues of proteins^21^. In the present study, the hepatic methylglyoxal levels were not significantly different between the ND and HFD groups, while GAPDH level was significantly higher in ND group, which was possibly due to high carbohydrate loading in ND group. The hepatic lipid accumulation in the HFD group was significantly higher than that in the ND group. Methylglyoxal might trigger oxidation of excess amount of fat in the liver. This assumption was supported by the significant decrease of lipid oxidation (TBARS) in liver with decrease of methylglyoxal via administration of WEC and PHA. Fat-derived malondialdehyde can also react with sulfhydryl groups in cysteine^39^. Increased amount of hepatic level of malondialdehyde (~50 μmol/kg) was higher than amount of methylglyoxal in the HFD group. Thus, higher amounts of malondialdehyde in liver of HFD group decreased free cysteine level compared to ND group. Shortage of cysteine promoted the reaction of short-chain aldehydes, such as methylglyoxal and malondialdehyde, with the sulfhydryl groups of proteins, including SOD-1 and GPX-1 and decreases their activity without decreasing the protein levels. Based on the findings of this study, we proposed a hypothesis that HFD-induced decreased GAPDH levels can maintain high methylglyoxal level despite low carbohydrate loading, which triggers the oxidation of fat deposited in the liver and consequently increases fat-derived malondialdehyde. Furthermore, malondialdehyde and methylglyoxal denature antioxidant enzymes by reacting with the sulfhydryl groups and induce liver damage. In contrast, free cysteine can protect against liver damage by reacting with methylglyoxal and malondialdehyde. On the basis of these findings, we proposed a novel approach to alleviate NAFLD involves the suppression of methylglyoxal formation by increasing GAPDH, which maintains the anti-oxidation activity in the fatty liver. To the best of our knowledge, increase of GAPDH by food compound has not been reported. The dose of PHA used in this study (10 μg/kg bodyweight) can be obtained through the consumption of some fermented foods^4–8^. Thus, food habits can improve liver function through GAPDH-methylglyoxal pathway.

Some in vitro studies have suggested that mild oxidative stress and hypoxia up-regulate the expression of GAPDH^40–42^, whereas high oxidative stress aggregates and inactivates GAPDH and consequently induces the apoptosis of cells^43,44^. Hypoxia-induced factor (HIF) plays an important role in the up-regulation of GAPDH^45^. The HIF level is regulated by several enzymes. There are ongoing studies to examine the effect of PHA on the expression of proteins involved in the regulation of GAPDH levels, such as HIF.

The administration of WEC (100 mg/kg bodyweight) decreased the plasma LDL-C levels and hepatic lipid oxidation, which are consistent with the results of a previous study^1^. In addition, effects of WEC on hepatic GAPDH, methylglyoxal, and cysteine are similar to PHA as summarized in Table 1. The consumption of WEC at a dose of 100 mg/kg bodyweight can achieve a PHA dose of ~1 μg/kg bodyweight^3^. Furthermore, WEC contains 440 mg/100 g cysteine and cystine as half cystine, which is similar level in HFD (430 mg/100 g) (Table 2). However, average consumption of HFD per day (~4 g/20 g body weight) was 2000 times higher than the dose of WEC (2 mg/20 g). Therefore, effect of supplementation of cystine and cysteine from WEC on liver functions could be neglected. Hence, PHA in WEC may contribute to the beneficial activities as summarized in Table 1 including LDL-C-lowering activity of WEC. The LDL-C-lowering activity exhibited by a low dose of phytochemical compounds is an interesting finding. The mechanism involved in the LDL-lowering effect of trace amounts of PHA has not been elucidated.

**Table 2:** Proximate composition and cystine content of animal diets (%).

	ND	HFD
Water	7.9	6.2
Crude protein	23.1	25.5
Crude fat	5.1	32
Crude ash	5.8	4.0
Crude fiber	2.8	2.9
Carbohydrate	55.3	29.4
Cystine	0.36	0.43

*ND* normal diet, *HFD* high-fat diet.

## Methods

### Materials and reagents

WEC was prepared and supplied by Sun Chlorella (Kyoto, Japan). WEC contained ~10 μg/g PHA in dry matter　and 4.4 mg/g half cystine (information from supplier). PHA, proteinase inhibitor cocktail, butylated hydroxytoluene (BHA), phosphate-buffered saline (PBS, pH 7.4), diethylenetriamine pentaacetic acid (DTPA), 1,1,3,3-tetraethoxypropane (TEP), thiobarbituric acid (TBA), 2,3-diaminonaphthalene (DAN), enhanced chemiluminescence (ECL) reagent, acetonitrile (high-performance liquid chromatography grade), and 4-vinylpyridine (4-VP) were purchased from Nacalai Tesque (Kyoto, Japan). Toronto Research Chemicals (Toronto, ON, Canada) provided 6-aminoquinolyl-*N*-hydroxysuccinimidyl carbamate (AccQ). Heparin sodium was obtained from Nipro (Osaka, Japan). Cell lysis reagent was obtained from Sigma-Aldrich (St. Louis, MO, USA). Tween 20 was purchased from Santa Cruz Biotechnology (Dallas, TX, USA). Rabbit immunoglobulin G (IgG) against mouse SOD-1 and GPX-1 were obtained from Abcam (Cambridge, UK). Mouse anti-GAPDH and β-actin monoclonal antibodies, were obtained from Proteintech (Chicago, IL, USA) and Santa Cruz Biotechnology. Goat horseradish peroxidase (HRP)-conjugated rabbit and mouse IgGs were obtained from Cell Signaling Technology (Danvers, MA, USA). Stable isotope-labeled L-cysteine (L-cysteine-¹³C_3_,^15^N) was purchased from Taiyo Nippon Sanso (Tokyo, Japan).

### Animal experiments

All animal experiments were performed according to the guidelines of the National Institutes of Health for the use of experimental animals. The experimental procedures were performed at the Louis Pasteur Center for Medical Research (Kyoto, Japan) and were approved by its Animal Care Committee (No. 20192).

Male C57BL/6 J mice (aged 7 weeks; bodyweight, 21–23 g) were purchased from Japan SLC (Shizuoka, Japan). The mice, which were housed in cages (three mice/cage), were maintained at 22 °C under a 12-h light/dark cycle with free access to rodent chow (Certified Diet MF, Oriental Yeast, Osaka, Japan) and tap water for 3 days. Subsequently, the mice were randomly divided into the following four groups (*n* = 6): ND group, fed on a standard diet; HFD group, fed on HFD; WEC group, fed on HFD and orally administered with WEC (100 mg/kg bodyweight) through drinking water; PHA group, fed on HFD and orally administered with PHA (10 μg/kg bodyweight) through drinking water. The proximate composition of animal diets is shown in Table 2. HFD (high-fat diet 32, CLEA Japan, Tokyo, Japan) comprises 32% crude fat with 60% of the calories derived from the fat. The amounts of WEC and PHA in drinking water were calculated based on the consumption of drinking water by mice in the previous 3 days. All mice received experimental diets for 12 weeks. The mice were then euthanized under isoflurane in the morning without fasting. The blood samples were collected from the inferior vena cava using a heparin-treated syringe. The plasma was separated by centrifuging the blood samples at 410 *g* for 5 min. The liver was excised and the blood in the liver was purged by infusing cold PBS into the portal vein. The plasma and liver samples were stored at −30 °C.

### Biochemical analyses

The analysis of plasma AST and ALT activities and the plasma levels of TG, TC, HDL-C, and LDL-C contents were outsourced to Oriental Yeast (Osaka, Japan). To determine the TG levels in the liver, liver samples (20–30 mg) were homogenized in 200 μL of isopropanol using a BioMasher II (Nippi, Tokyo, Japan). The homogenates were centrifuged at 1100 × *g* for 10 min. The supernatant was subjected to a TG assay using the TG E-test Wako kit (Wako, Osaka, Japan).

### TBARS assay

The working solutions of DTPA (2 mM) and BHA (10%) were prepared in 1 M sodium acetate buffer (pH 3.5) and methanol, respectively. To prepare the TBA solution, 0.1 g TBA was mixed with 5 mL of DTPA solution, 45 mL of hot distilled water, and 100 μL of BHA solution. The stock solution (2 mM) of TEP (a precursor of malondialdehyde) was prepared by adding 4.8 μL TEP to 10 mL of methanol. The standards (2–50 μM) were prepared from the stock solution of TEP. The liver tissues were homogenized in nine volumes (v/w) of 1.15% KCl solution. The homogenates were centrifuged at 2000 × *g* for 1 min. The supernatant or standard solution (25 μL) was mixed with TBA solution (100 μL), vortexed, and heated at 95 °C for 60 min. The reaction was terminated by cooling the reaction mixture on ice for 5 min. The reactants were centrifuged at 14,200 × *g* for 10 min and the absorbance at 515 nm of the supernatant was measured.

### Evaluation of hepatic SOD-like and GPX-like activities

Liver samples were homogenized in five volumes (v/w) of 10 mM Tris-HCl buffer (pH 7.4) containing 0.25 M sucrose and 1 mM ethylenediaminetetraacetic acid (EDTA) using a BioMasher II. The homogenates were centrifuged at 10000 × *g* for 60 min and the total SOD-like activity in the supernatant was assayed using a WST-1 SOD assay kit (Dojindo, Kumamoto, Japan). To examine the SOD-like activity in the low molecular weight compounds in the extract, the liver tissues were homogenized with one volume (v/w) of PBS using a BioMasher II. The homogenate was mixed with six volumes (v/w) of ethanol. The resultant suspension was centrifuged at 14200 × *g* for 10 min. The SOD-like activity in the supernatant was assayed. The liver extract used for the TBARS assay was also used for assaying GPX-like activity. The GPX-like activity in the supernatant was assayed using a glutathione peroxidase activity colorimetric assay kit (BioVision, Milpitas, CA, USA).

### Determination of hepatic methylglyoxal levels

The methylglyoxal level was determined using the liquid chromatography-tandem mass spectrometry system (LC-MS/MS) after derivatization with DAN following the methods of Han et al. with minor modifications^46^. Liver samples were homogenized with one volume (v/w) of PBS using a BioMasher II. The homogenate was mixed with six volumes (v/w) of ethanol and the suspension was centrifuged at 14200 × *g* and 4 °C for 10 min. The supernatant (10 µL) was incubated with 50 μL of 0.1 % (w/v) DAN solution at 50 °C for 1 h. The reactants were mixed with 500 μL ethyl acetate and vortexed. The ethyl acetate layer (400 μL) was collected and evaporated. The residue was dissolved in a 30% aqueous acetonitrile solution. Aliquots of the solution were clarified by passing it through a Cosmonice filter W (Nacalai Tesque). DAN derivatives of methylglyoxal were quantified using an LC-MS/MS system equipped with an ODS-3 column in multiple reaction monitoring (MRM) mode. The MRM condition for the DAN derivatives were optimized using LabSolutions Version 5.65. A binary linear gradient, with 0.1% formic acid (solvent A) and 0.1% formic acid containing 80% acetonitrile (solvent B), was used at a flow rate of 0.2 mL/min. The gradient program was as follows: 0–15 min, 0–100% B; 15–20 min, 100% B; 20–20.1 min, 100–0% B; 20.1–30 min, 0% B. The column temperature was maintained at 40 °C.

### Western blotting

The liver samples (~50 mg) were homogenized with 300 μL of cell lysis reagent containing 1% proteinase inhibitor using a BioMasher II. The homogenate was centrifuged at 12000 × *g* and 4 °C for 15 min. The protein concentration in the supernatant was measured using a BCA protein assay kit (Thermo Scientific, Rockford, IL, USA) and the concentration was adjusted to 10 μg/15 μL with the same buffer. All samples were mixed with the same volume of pre-stained marker (Prestained XL-Ladder, Integrale, Tokushima, Japan) to ensure the consistency of loading volume and transfer efficiency. The proteins were subjected to sodium dodecyl sulfate-polyacrylamide gel electrophoresis using a 12.5% gel. The resolved proteins were transferred to a polyvinylidene difluoride membrane (0.45 μm; GE Healthcare Life Sciences, Chicago, IL, USA) using a semi-dry blotting apparatus (WSE 4020, Atto, Tokyo, Japan). The band intensity of pre-stained markers was quantified using ImageJ 1.52a. The membranes were then blocked with 4% Block Ace solution (Megmilk Snow Brand, Sapporo, Japan) at room temperature (25 °C) for 30 min and washed five times with 50 mM Tris-HCl buffer (pH 7.5) containing 2.68 mM KCl, 137 mM NaCl, and 0.05% (v/v) Tween 20 (TBST) for 5 min at room temperature. Primary antibodies against SOD-1, GPX-1, GAPDH, and β-actin were diluted to 1:5000, 1:5000, 1:8000 and 1:1000 with 0.4% Block Ace solution containing 0.05% (v/v) Tween 20, respectively. After overnight incubation with the primary antibody, the membranes were washed with TBST for 5 min (5 times). HRP-conjugated secondary antibodies against rabbit or mouse IgG were diluted to 1:10000 and 1:12500 with 0.4% Block Ace containing 0.05% (v/v) Tween 20, respectively. The membranes were incubated with secondary antibodies for 1 h, followed by washing with TBST for 5 min (5 times) at room temperature. The membranes were soaked with ECL reagent for 1 min. Immunoreactive bands were detected using a Lumino Graph I (Atto). To correct loading volume and transfer efficiency, chemiluminescence intensity of each　band was divided by the band intensity of pre-stained markers near the target protein　(35 kDa for GAPDH and β-actin, 30 kD for SOD-1 and GPX-1). The ratio was used as band intensity. To compare band intensity of specific protein in different gels, the band intensity of each gel was normalized by the band intensity of common sample in HFD group, which was analyzed by all gels.

### Determination of hepatic levels of reduced and oxidative forms of cysteine

The same sample used for the determination of methylglyoxal level was used for the determination of cysteine level. The supernatant (37.5 μL) was mixed with same volume of an internal standard (0.1 mM of L-cysteine-¹³C_3_,^15^N) and 22.5 μL of 75% ethanol or 2 mM DTT in 75% ethanol solution for 1 min to detect the levels of reduced cysteine and total cysteine, respectively. Next, the sample was incubated with 4-VP (2.5 μL) at 37 °C for 2 h. Ethyl acetate (500 μL) and distilled water (100 μL) were added to the reactants and vortexed. The water layer (50 μL) was collected and evaporated under vacuum. Sodium borate buffer (50 mM; pH 8.8; 80 μL) and 0.3% AccQ acetonitrile solution (20 μL) were added to the residue. The resultant solution was kept at 50 °C for 10 min. The reactant was clarified by passing it through a Cosmonice filter W. The resultant cysteine derivatives with 4-VP and AccQ were quantified using an LC-MS/MS system equipped with an ODS-3 column in MRM mode. The MRM conditions for the cysteine and cysteine-¹³C_3_,^15^N derivatives were optimized using LabSolutions Version 5.65. A binary linear gradient, with 0.1% formic acid (solvent A) and 0.1% formic acid containing 80% acetonitrile (solvent B), was used at a flow rate of 0.2 mL/min. The gradient program was as follows: 0–15 min, 0–50% B; 15–20 min, 50–100% B; 20–25 min, 100% B; 25–25.1 min, 100–0% B; 25.1–35 min, 0% B. The column temperature was maintained at 40 °C. The levels of cysteine in the extract were estimated using the following equation: [(peak area of cysteine derivative in the sample/peak area of the　internal standard) × concentration of the internal standard].

### Statistical analyses

The results are presented as mean ± standard deviation. The means was analyzed using one-way analysis of variance, followed by Dunnett’s test for multiple comparisons vs. HFD group. The differences were considered significant at *p* < 0.05 and to have a certain tendency at 0.05 <*p* < 0.1. GraphPad Prism 7 software (GraphPad Software, San Diego, CA, USA) was used for statistical analyses.

### Reporting summary

Further information on research design is available in the Nature Research Reporting Summary linked to this article.

## Supplementary information

Supplementary Information

Reporting Summary

## Data Availability

The data supporting the findings reported herein are available on reasonable request from the corresponding author.
