# Supplementary material for: Phenethylamine in chlorella alleviates high-fat diet-induced mouse liver damage by regulating generation of methylglyoxal
Source: NPJ Sci Food. 2021 Jul 23;5:22. doi: 10.1038/s41538-021-00105-3 (PMC8302609; doi:10.1038/s41538-021-00105-3)
Supplement: Supplementary file 1 — Supplementary Information [file 41538_2021_105_MOESM1_ESM.pdf]

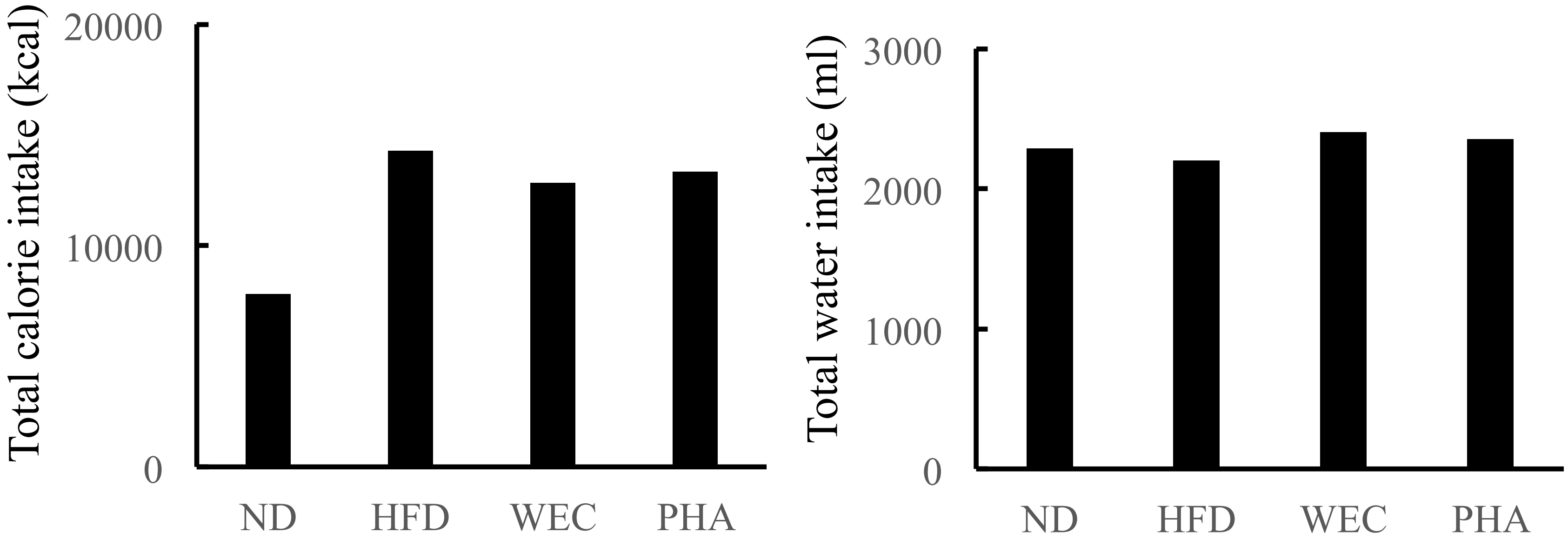

Figure S1. Total calorie and water intake of mice of the normal diet-fed (ND), high-fat diet-fed (HFD), *Chlorella pyrenoidosa* water extract-treated (WEC), and phenethylamine-treated (PHA) groups.

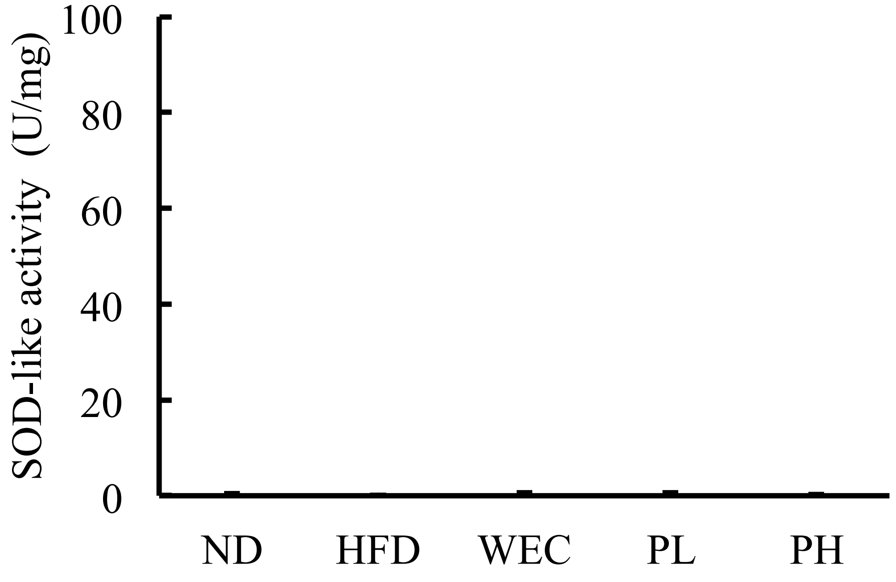

Figure S2. SOD-like activity in low molecular weight compounds in liver extract.
Refer Figure S1 legend for abbreviation of each group.

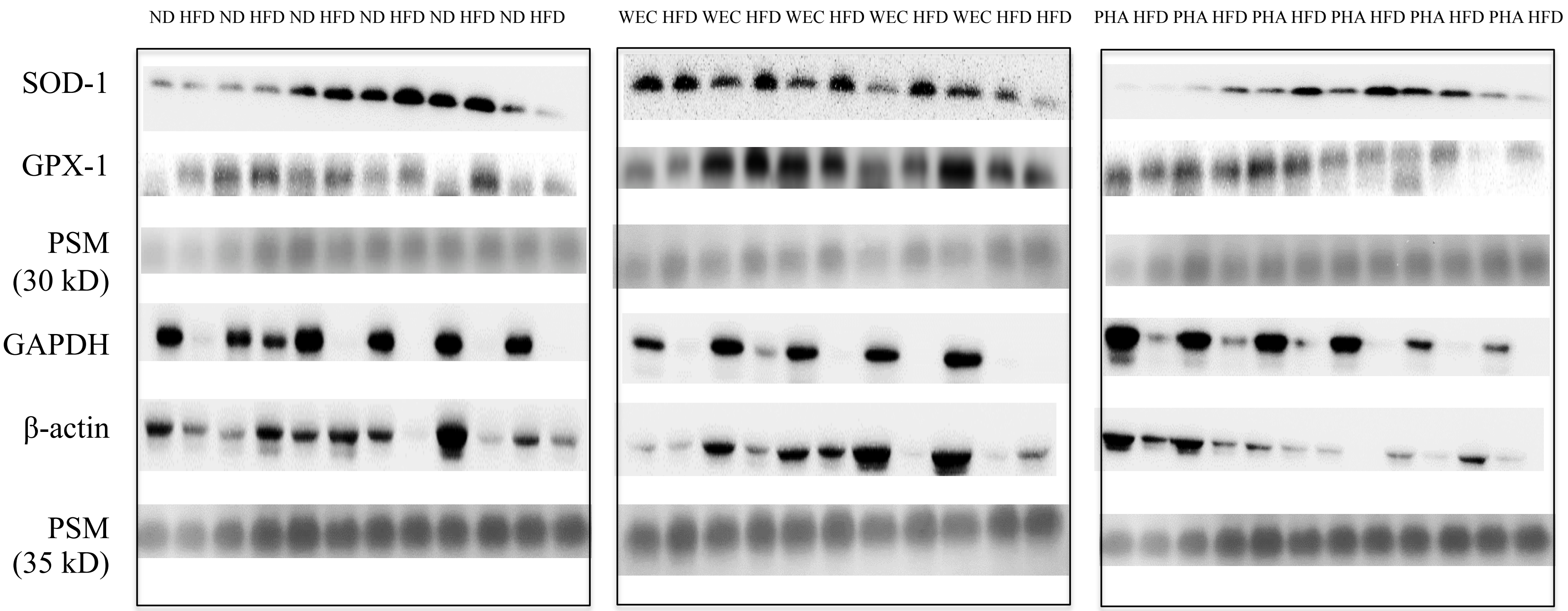

Figure S3. All images of western blots.
Representative three lane for each group are shown in Figure 5 and Supplementary Figure S4.
Refer Figure S1 legend for abbreviation of each group.
PSM, pre-stained marker.

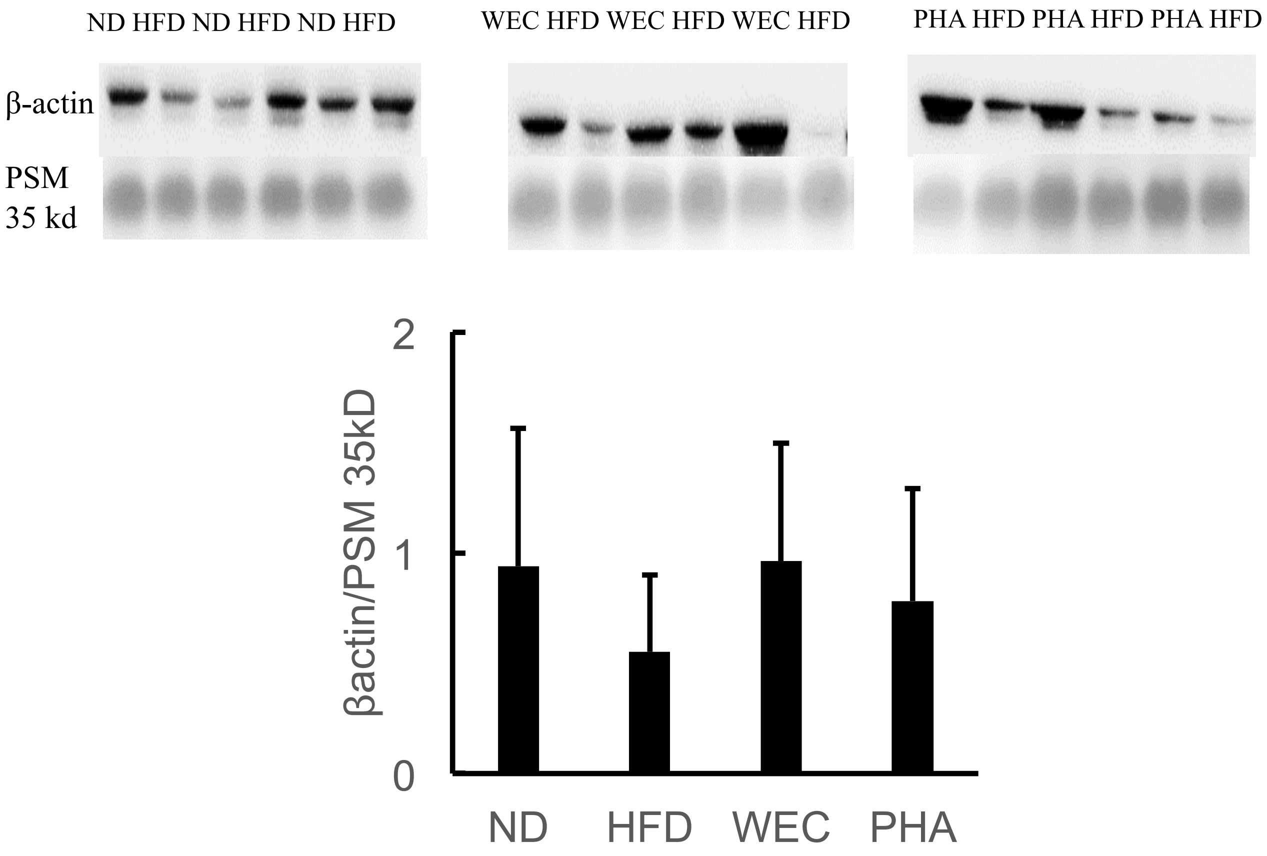

Figure S4. Hepatic β-actin levels of mice in each group.
See Materials and Methods section for experimental details.
Refer Figure S1 legend for abbreviation of each group.

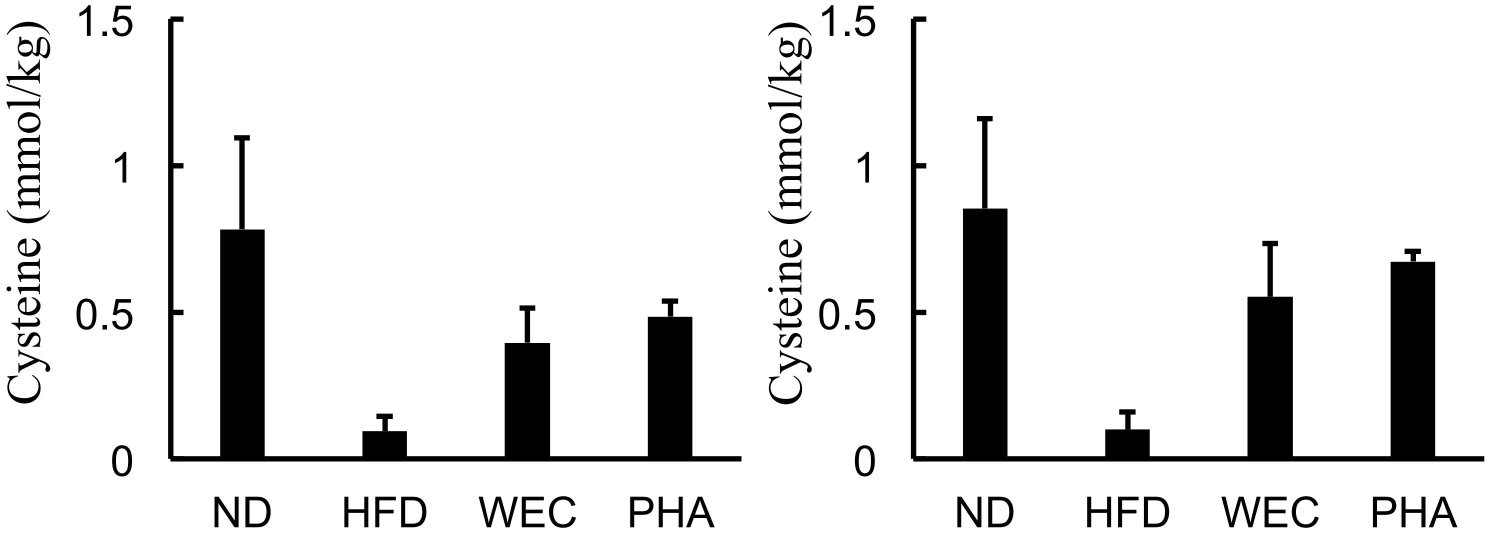

Figure S5. Hepatic total (left) and reduced cysteine (right) levels of each group. Refer Figure S1 legend for abbreviation of each group.
