# Supplementary material for: Phenethylamine in chlorella alleviates high-fat diet-induced mouse liver damage by regulating generation of methylglyoxal
Source: NPJ Sci Food. 2021 Jul 23;5:22. doi: 10.1038/s41538-021-00105-3 (PMC8302609; doi:10.1038/s41538-021-00105-3)
Supplement: Supplementary file 2 — Reporting Summary [file 41538_2021_105_MOESM2_ESM.pdf]

nature portfolio

Corresponding author(s): kenji sato
Last updated by author(s): Jul 1, 2021

Reporting Summary

Nature Portfolio wishes to improve the reproducibility of the work that we publish. This form provides structure for consistency and transparency in reporting. For further information on Nature Portfolio policies, see our Editorial Policies and the Editorial Policy Checklist.

Statistics

For all statistical analyses, confirm that the following items are present in the figure legend, table legend, main text, or Methods section.

n/a	Confirmed	
☐	☒	The exact sample size (*n*) for each experimental group/condition, given as a discrete number and unit of measurement
☐	☒	A statement on whether measurements were taken from distinct samples or whether the same sample was measured repeatedly
☐	☒	The statistical test(s) used AND whether they are one- or two-sided<br>*Only common tests should be described solely by name; describe more complex techniques in the Methods section.*
☐	☒	A description of all covariates tested
☐	☒	A description of any assumptions or corrections, such as tests of normality and adjustment for multiple comparisons
☐	☒	A full description of the statistical parameters including central tendency (e.g. means) or other basic estimates (e.g. regression coefficient) AND variation (e.g. standard deviation) or associated estimates of uncertainty (e.g. confidence intervals)
☐	☒	For null hypothesis testing, the test statistic (e.g. *F*, *t*, *r*) with confidence intervals, effect sizes, degrees of freedom and *P* value noted<br>*Give P values as exact values whenever suitable.*
☒	☐	For Bayesian analysis, information on the choice of priors and Markov chain Monte Carlo settings
☒	☐	For hierarchical and complex designs, identification of the appropriate level for tests and full reporting of outcomes
☒	☐	Estimates of effect sizes (e.g. Cohen's *d*, Pearson's *r*), indicating how they were calculated

Our web collection on statistics for biologists contains articles on many of the points above.

Software and code

Policy information about availability of computer code

Data collection	LabSolutions Version 5.65. for determination based on LC-MS/MS. ImageJ 1.52a and Lumino Graph I for determination based on Western blot analysis.
Data analysis	GraphPad Prism 7 software (GraphPad Software)

For manuscripts utilizing custom algorithms or software that are central to the research but not yet described in published literature, software must be made available to editors and reviewers. We strongly encourage code deposition in a community repository (e.g. GitHub). See the Nature Portfolio guidelines for submitting code & software for further information.

Data

Policy information about availability of data

All manuscripts must include a data availability statement. This statement should provide the following information, where applicable:
- Accession codes, unique identifiers, or web links for publicly available datasets
- A description of any restrictions on data availability
- For clinical datasets or third party data, please ensure that the statement adheres to our policy

The data supporting the findings reported herein are available on reasonable request from the corresponding author. Data are not stored in publicity available datasets.

Field-specific reporting

Please select the one below that is the best fit for your research. If you are not sure, read the appropriate sections before making your selection.

☒ Life sciences ☐ Behavioural & social sciences ☐ Ecological, evolutionary & environmental sciences

For a reference copy of the document with all sections, see nature.com/documents/nr-reporting-summary-flat.pdf

Life sciences study design

All studies must disclose on these points even when the disclosure is negative.

Sample size	We carried preliminary experiments using 6 animals (3 animal in same cage) for each group under similar condition. We got statistically significance. Thus, we chose 6 animals for each group.
Data exclusions	No data exclusion.
Replication	As mentioned before, we carried preliminary experiment and obtained similar results.
Randomization	Mice with same age and similar body weigh were obtained from same supplier. Subsequently, the mice were randomly divided into the following four groups (n = 6).
Blinding	For the LC-MS/MS analysis, samples were randomly analyzed. However, for western blot analysis, samples were arranged control, treated, control, treated...to make easy comparison. Thus, investigator could realize which sample. However, image data are presented in Figure to confirm rationality.

Reporting for specific materials, systems and methods

We require information from authors about some types of materials, experimental systems and methods used in many studies. Here, indicate whether each material, system or method listed is relevant to your study. If you are not sure if a list item applies to your research, read the appropriate section before selecting a response.

Materials & experimental systems

n/a	Involved in the study	
☐	☒	Antibodies
☒	☐	Eukaryotic cell lines
☒	☐	Palaeontology and archaeology
☐	☒	Animals and other organisms
☒	☐	Human research participants
☒	☐	Clinical data
☒	☐	Dual use research of concern

Methods

n/a	Involved in the study	
☒	☐	ChIP-seq
☒	☐	Flow cytometry
☒	☐	MRI-based neuroimaging

Antibodies

Antibodies used	Rabbit immunoglobulin G (IgG) against mouse SOD-1 (ab183881) and GPX-1 (ab22604) were obtained from Abcam (Cambridge, UK). Mouse anti-GAPDH (60004-1-Ig) and β-actin (sc-81178) monoclonal antibodies, were obtained from Proteintech (Chicago, IL, USA) and Santa Cruz Biotechnology. Goat horseradish peroxidase (HRP)-conjugated rabbit (#7074) and mouse IgGs (#7076) were obtained from Cell Signaling Technology (Danvers, MA, USA).
Validation	On the manufactuer's website.

Animals and other organisms

Policy information about studies involving animals; ARRIVE guidelines recommended for reporting animal research

Laboratory animals	Male C57BL/6J mice (aged 7 weeks; bodyweight, 21–23 g) were purchased from Japan SLC (Shizuoka, Japan).
Wild animals	This study did not use wild animal.
Field-collected samples	This study did not use sample collected from field.
Ethics oversight	All animal experiments were performed according to the guidelines of the National Institutes of Health for the use of experimental animals. The experimental procedures were performed at the Louis Pasteur Center for Medical Research (Kyoto, Japan) and were approved by its Animal Care Committee (No. 20192).

March 2021

Note that full information on the approval of the study protocol must also be provided in the manuscript.
